# Spatiotemporal reconstruction and transmission dynamics during the 2016–17 H5N8 highly pathogenic avian influenza epidemic in Italy

**DOI:** 10.1111/tbed.13420

**Published:** 2019-12-01

**Authors:** William T. Harvey, Paolo Mulatti, Alice Fusaro, Francesca Scolamacchia, Bianca Zecchin, Isabella Monne, Stefano Marangon

**Affiliations:** ^1^ Boyd Orr Centre for Population and Ecosystem Health Institute of Biodiversity, Animal Health and Comparative Medicine College of Medical, Veterinary and Life Sciences University of Glasgow Glasgow UK; ^2^ Istituto Zooprofilattico Sperimentale delle Venezie Legnaro (Padua) Italy

**Keywords:** avian influenza, H5N8 subtype, infectious disease outbreaks, infectious disease reservoirs, phylogeny, phylogeography

## Abstract

Effective control of avian diseases in domestic populations requires understanding of the transmission dynamics facilitating viral emergence and spread. In 2016–17, Italy experienced a significant avian influenza epidemic caused by a highly pathogenic A(H5N8) virus, which affected domestic premises housing around 2.7 million birds, primarily in the north‐eastern regions with the highest density of poultry farms (Lombardy, Emilia‐Romagna and Veneto). We perform integrated analyses of genetic, spatiotemporal and host data within a Bayesian phylogenetic framework. Using continuous and discrete phylogeography, we estimate the locations of movements responsible for the spread and persistence of the epidemic. The information derived from these analyses on rates of transmission between regions through time can be used to assess the success of control measures. Using an approach based on phylogenetic–temporal distances between domestic cases, we infer the presence of cryptic wild bird‐mediated transmission, information that can be used to complement existing epidemiological methods for distinguishing transmission within the domestic population from incursions across the wildlife–domestic interface, a common challenge in veterinary epidemiology. Spatiotemporal reconstruction of the epidemic reveals a highly skewed distribution of virus movements with a high proportion of shorter distance local movements interspersed with occasional long‐distance dispersal events associated with wild birds. We also show how such inference be used to identify possible instances of human‐mediated movements where distances between phylogenetically linked domestic cases are unusually high.

## 1 INTRODUCTION

In 2016–17, a highly pathogenic avian influenza (HPAI) epidemic caused by A(H5N8) viruses of clade 2.3.4.4 occurred in 29 European Countries. This was the largest HPAI epidemic ever recorded in Europe in terms of number of poultry outbreaks, wild bird deaths recorded and geographical extent (Adlhoch et al., 2018; Brown, Kuiken, et al., 2017; Brown, Mulatti, et al., 2017). Between October 2016 and December 2017, approximately 1,500 cases were identified in wild birds and more than 1,200 outbreaks were reported in poultry across Europe, with frequent primary incursions affecting all productive sectors, ranging from backyard/rural to industrial. Secondary spread between domestic premises was also detected, most commonly associated with domestic waterfowl species (Brown, Kuiken, et al., 2017; Brown, Mulatti, et al., 2017).

The first HPAI cases in Italy were identified in late December 2016 with two A(H5N8) and two A(H5N5) HPAI viruses confirmed in dead wild birds found in north‐eastern Italy between 30 December 2016 and 23 January 2017 (Fusaro et al., 2017). While H5N5 subtype viruses were not further detected, A(H5N8) HPAI spread widely in northern Italy leading to several cases in both domestic poultry and wild birds (Fusaro et al., 2017). In contrast to the rest of Europe, Italy experienced two distinct epidemic waves during 2016–2017 which presented different characteristics (Mulatti et al., 2018). The first wave which comprised 16 cases confirmed in poultry and five in wild birds (excluding two A(H5N5) cases) started in late December 2016 and continued until late May 2017 following an atypical epidemic curve, with sporadic weekly reported cases and a limited involvement of wild birds in discordance with observations in other European Countries (Globig et al., 2017; Mulatti et al., 2018; Verhagen et al., 2017). Most cases were detected at the periphery of the most densely populated poultry area (DPPA) in the northern Italian regions of Veneto, Lombardy and Emilia‐Romagna. The second epidemic wave started in the second half of July 2017 and ended by mid‐December of the same year (Brown, Kuiken, et al., 2017; Mulatti et al., 2018). Sixty‐seven outbreaks were observed in poultry farms, mainly fattening turkey premises, while only seven cases were confirmed in wild birds. During the second wave, the virus spread to the DPPA in the Po Valley, where most of the cases were located. Sporadic, isolated outbreaks were also notified in Piedmont, and Lazio, in western and central Italy, respectively. The second epidemic wave presented a classic occurrence pattern for an infectious disease spreading in a densely populated area, with evidence of lateral spread leading to clusters of interconnected outbreaks (Mulatti et al., 2018).

For rapidly evolving viruses, the time scale of epidemic spread often enables integrated reconstruction of both the evolutionary history and spatial processes underlying an epidemic (Dellicour, Rose, & Pybus, 2016; Lemey, Rambaut, Drummond, & Suchard, 2009; Pybus, Tatem, & Lemey, 2015). Phylogeographic inference has become a commonly used technique for the study of virus dispersal over the course of an epidemic in either discrete (Bedford et al., 2015; Brunker et al., 2018; Dudas et al., 2017; Hall, Knowles, Wadsworth, Rambaut, & Woolhouse, 2013; Lemey et al., 2009; Trewby, Nadin‐Davis, Real, & Biek, 2017) or continuous space (Brunker et al., 2018; Dellicour, Baele, et al., 2018; Dellicour, Vrancken, Vrancken, Trovão, Fargette, & Lemey, 2018; Jacquot, Nomikou, Palmarini, Mertens, & Biek, 2017). Although the grouping of locations implicit in discrete phylogeography is often arbitrary, the boundaries used may correspond to borders around which control measures are structured and these analyses can be useful for quantifying transmission rates across national or international borders (Dudas et al., 2017; Trewby et al., 2017). Continuous phylogeography which models change in latitude and longitude as a diffusion process along branches of the phylogeny can provide a more detailed vision of the dispersal process, though requires more comprehensive location data and assumes the dispersal process maintain a relationship with geographical distance (Dellicour, Vrancken, et al., 2018), an assumption that may generally not be the case for human influenza viruses which have been shown to follow air transportation migration routes at a global scale (Lemey et al., 2014; Bedford et al., 2015). Owing to this in part, discrete phylogeography has often been applied in the study of influenza viruses in general (Bedford et al., 2015; Lemey et al., 2009; Lu, Leigh Brown, & Lycett, 2017; Lu, Lycett, & Brown, 2014; Lycett et al., 2012; Magee, Beard, Suchard, Lemey, & Scotch, 2015; The Global Consortium for H5N8 and Related Influenza Viruses, 2016). The discrete trait modelling framework used for discrete phylogeography has also been used to reconstruct ancestral hosts across phylogenies for a range of viruses, including HPAI H5N8 (Black, Breyta, Bedford, & Kurath, 2016; Dudas, Carvalho, Rambaut, & Bedford, 2018; Hall et al., 2013; Lu et al., 2014; The Global Consortium for H5N8 and Related Influenza Viruses, 2016; Trovão, Suchard, Baele, Gilbert, & Lemey, 2015). Such reconstructions enable various insights, for example, on source–sink dynamics and the quantification of the proportion of evolution occurring within different host species (Dudas et al., 2018) or the support for epidemiological links between host species associated with outbreaks of avian influenza (Lu et al., 2014).

Avian influenza outbreaks are typically epidemiologically complex in nature, as they may involve multiple wild bird species that vary in spatial ecology and clinical disease severity, and domestic incursions may affect various species that differ in housing, susceptibility and connectedness. Possible routes of transmission include migratory and local movements of wild birds, farm‐to‐farm transmission between holdings in close proximity and human‐mediated transmission involving transport of infected birds or sharing of vehicles or personnel between holdings (Brown, Kuiken, et al., 2017; Brown, Mulatti, et al., 2017; Mulatti et al., 2018). The integration of various data types using phylodynamic analyses can shed insight into transmission dynamics and the processes shaping an epidemic. For example, the contribution of wild birds, different poultry species and production types to the onwards transmission and the routes of transmission responsible for geographical expansion of an epidemic may be elucidated. Ultimately, a better understanding of pathogen dispersal and transmission during the course of an epidemic may help to assess existing prevention and intervention measures and shape future strategies.

In this paper, we present integrated phylogenetic and phylodynamic analyses of genetic, geographical and host data to reveal features of the 2016–17 A(H5N8) HPAI epidemic in Italy. We reconstruct the geographical pattern of pathogen dispersal using both continuous and discrete phylogeographic approaches. We also reconstruct host types across the phylogeny describing the epidemic to allow significant host type‐to‐host type routes of transmission to be identified. To account for likely under‐sampling of the epidemic in wild birds, we infer the presence of cryptic wild bird‐mediated transmission across the phylogeny through consideration of evolutionary and geographical distances between observed cases. Transition rates between geographical regions and host types are quantified over the duration of the epidemic. Finally, relationships between host types and reconstructed movement distances are explored allowing inferences on the production types associated with virus dispersal to be determined.

## 2 MATERIALS AND METHODS

### 2.1 Data

Virus sequences and epidemiological data for 83 domestic cases and 12 wild birds were analysed. Epidemiological investigations, the collection of dead birds and sequencing were carried out as described in our previous work (Fusaro et al., 2017; Mulatti et al., 2018). Virus sequences have been deposited in the GISAID EpiFlu Database (https://platform.gisaid.org/) in association with the aforementioned studies. The accession numbers of sequences used for analysis in the paper are as follows: EPI1261344‐ EPI1261994, EPI961493‐EPI961507, EPI961509‐EPI961522, EPI961524‐EPI961527, EPI1040223‐EPI1040238, EPI1081918‐EPI1081924 and EPI1081966‐EPI1081973. For each Case ID, date, host type, geographical region and province are detailed in Table S1.

### 2.2 Phylogenetic analysis

Time‐resolved phylogenetic trees were reconstructed using BEAST v1.10.4 (Suchard et al., 2018). Sequence evolution was modelled using a general time reversible model with a proportion of invariant sites, a gamma distribution describing among‐site rate variation with four categories estimated from the data (GTR + I + Γ_4_) and allowing for rates of change at the third codon position to differ relative to the first two. This model was implemented with a relaxed molecular clock with branch rates drawn from a lognormal distribution (Drummond, Ho, Phillips, & Rambaut, 2006) and a minimally constrained Bayesian skyline demographic model (Drummond, Rambaut, Shapiro, & Pybus, 2005). Four runs of 50,000,000 steps were sampled every 5,000 steps. Convergence and mixing properties were inspected using Tracer v1.7.1 (https://github.com/beast-dev/tracer/releases), and after removing 10% of samples as burn‐in, chains were combined at a reduced sampling rate of 20,000 producing a posterior sample of 9,000 trees. Maximum clade credibility (MCC) trees were identified using TreeAnnotator v1.10.4 (Suchard et al., 2018), and phylogenies were visualized using the ggtree R package (Yu, Smith, Zhu, Guan, & Lam, 2016).

### 2.3 Continuous phylogeography

Spatial diffusion was mapped to viral phylogenies using the continuous phylogeographic framework described by Lemey, Rambaut, Welch, and Suchard, (2010) implemented in BEAST. A homogenous Brownian diffusion model, which assumes that the rate of spatial diffusion does not vary significantly across the phylogeny, was compared with relaxed random walk (RRW) models (Lemey et al., 2010) which include branch‐specific scaling factors drawn from an underlying distribution (either Cauchy, gamma or lognormal). Brownian and RRW models describing the distribution of spatial diffusion across the phylogeny were compared using AICM. Reconstructed dates and geographical coordinates at internal nodes of the phylogeny were extracted, and per‐branch dispersal distances were calculated from geographical coordinates by converting latitude and longitude to radians and applying the spherical law of cosines with the radius of the Earth specified as 6,371 km. For visualization, branches were dated at the midpoint between the dates estimated for the nodes they connect.

### 2.4 Discrete trait analysis

Rates of transition between geographical regions and between host types were estimated in BEAST by inferring models of discrete trait evolution using an asymmetric model that allows the rate of transition between any given pair of traits to differ depending on the direction (Lemey et al., 2009). The number of sequences in each discrete state for the both the geographical and host analyses is stated in Table S2. Bayesian stochastic search variable selection (BSSVS) was used to identify phylogenetically robust transitions by assigning a binary indicator variable to each transition which was sampled by MCMC. Transitions included in the model (indicator variable = 1) in more than 50% of MCMC samples in the combined sample were considered significant. For significant transitions, conditional mean rates were calculated (i.e. mean when included). To examine transmission events through time, the time and inferred geographical region, or host type, at each node of the phylogeny across a posterior set of trees was extracted. Transmission event timing was estimated as the midpoint between the dates associated with each end of a given branch. Times were collated into fortnightly bins (1/26 of a year) to produce distributions of transmission events through time following Lycett et al. (2012).

### 2.5 Inference of cryptic wild bird‐mediated transmission

Phylogenetic, or patristic, distances between viruses positioned in time‐scaled phylogenetic trees were calculated and averaged across a posterior sample of 100 trees. For each domestic case, a putative domestic ancestor was identified as the domestic case recorded on a previous date with the shortest patristic distance. Each domestic case was thus associated with an evolutionary distance to its putative domestic ancestor. The minimum and maximum distances to putative domestic ancestors were used to define a range of threshold phylogenetic distances. Using thresholds at intervals of 0.005 within this range, a post‐order traversal that proceeds from the tips towards the root of the tree was used to infer either wild bird or domestic host type at each internal node of the phylogeny. Internal nodes with both descendants being domestic cases with evolutionary distances to their putative domestic ancestor below this threshold were inferred to be domestic, and otherwise, a wild bird host was inferred. At internal nodes deeper in the tree, domestic host type was inferred if both descendant nodes were inferred to be domestic, otherwise wild bird was inferred.

Domestic cases were classified as being the result of primary (wild bird to domestic) or secondary (domestic to domestic) transmission depending on whether the internal node from which they were descended was inferred to be wild bird or domestic using the above methodology. Using a range of phylogenetic distance thresholds, the classification of cases made using this approach was compared with the reported classification of primary and secondary cases made using established epidemiological methods (Mulatti et al., 2018). For each value used as a threshold phylogenetic distance, the sensitivity and precision with which secondary cases reported by Mulatti et al. were calculated. From these measures, *F*‐scores which balance sensitivity and precision were calculated. Using thresholds that resulted in a reasonable level of accordance (*F*_1.5_ > 0.8 and specificity >0.7), the proportion of threshold values at which each internal node was estimated to be wild bird was calculated. Rates of transmission between and within wild and domestic birds through time were calculated as described in the above discrete trait analysis section. To combine the results of the discrete trait reconstruction of host type and this analysis, internal nodes retained their host type inferred using the discrete trait model unless a wild bird host was inferred using the distance‐based approach. Rates of transition between each of the host categories considered in the discrete trait analysis (backyard, broiler, layer, turkey, domestic waterfowl and wild bird) were calculated and averaged across a posterior sample of 100 trees. Rates greater than one when averaged across the posterior set of phylogenies were considered significant and plotted in a network map.

## 3 RESULTS

**Figure 1:**
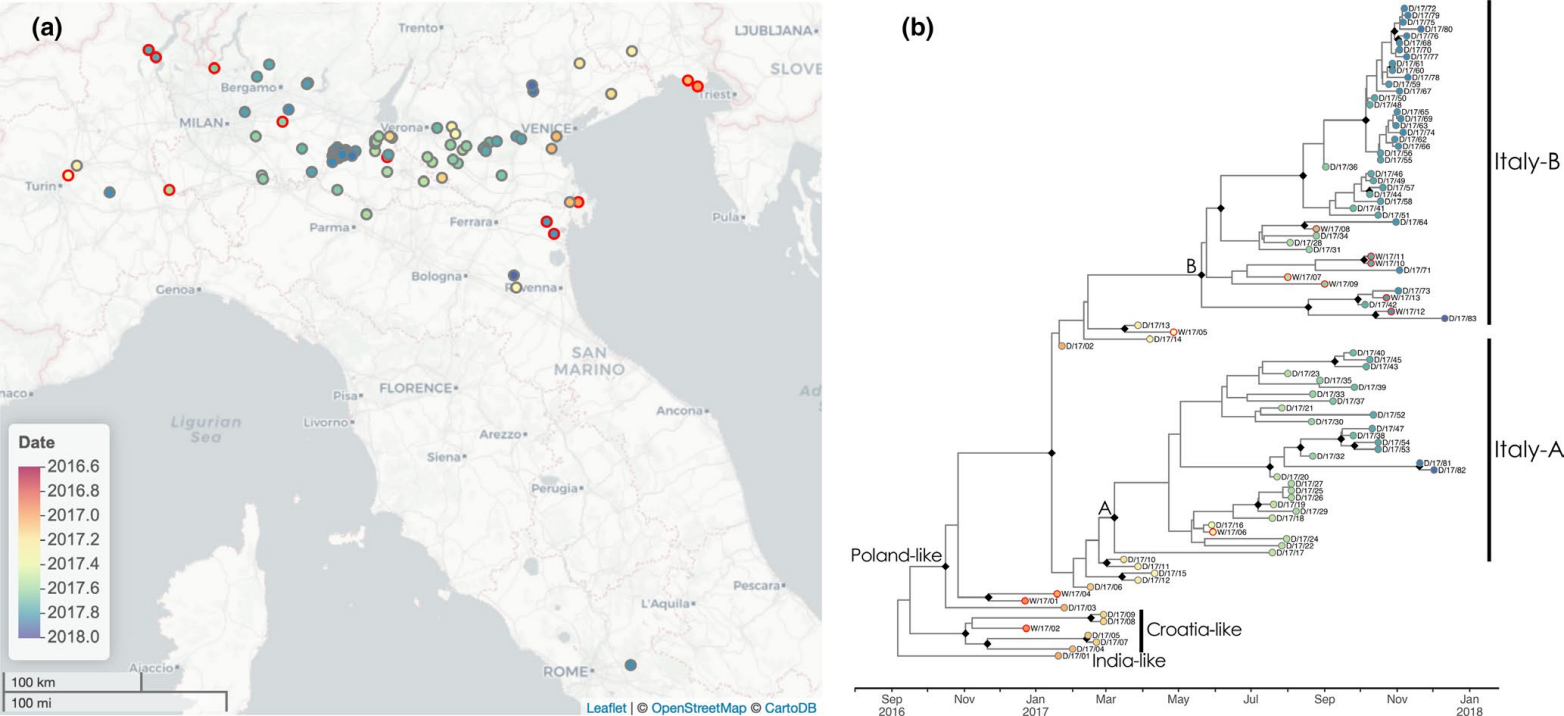
The spatial and phylogenetic structure of 95 influenza A(H5N8) viruses from 83 domestic cases and 12 wild birds sampled between December 2016 and December 2017. (a) Locations of domestic (grey rings) and wild bird (red rings) cases within Italy coloured by date of sampling. Map tiles by Carto, under CC BY 3.0. Data by OpenStreetMap, under ODbL. (b) Time‐scaled maximum clade credibility phylogenetic reconstruction of HA gene sequences, with posterior support for internal nodes >0.8 indicated by black diamonds

A set of time‐scaled phylogenetic trees were estimated from haemagglutinin (HA) gene sequences of 95 influenza A(H5N8) viruses sampled during the 2016–17 highly pathogenic avian influenza epidemic in Italy were reconstructed using BEAST. To enable evolutionary investigation of transmission dynamics throughout the epidemic, the location and host type from which samples were taken were incorporated into this analysis as both continuous and discrete traits reconstructed at internal nodes of these phylogenies. Sampling locations are shown in Figure 1a, while the MCC phylogenetic tree identified from the BEAST analysis is shown in Figure 1b. The date of the root of the tree was dated to the second half of 2016 with a point estimate of 6 September 2016 and a 95% highest posterior density interval (HPD) of 28 July to 4 November. The first epidemic wave has been described as being characterized by the circulation of three genetically distinct strains, (labelled India‐, Poland‐ and Croatia‐like in Figure 1b after H5N8‐like viruses A/painted stork/India/10CA03/2016, A/wild duck/Poland/82A/2016, and A/mute swan/Croatia/70/2016) (Fusaro et al., 2017). The recent date estimated for the HA sequences analysed compared with estimates derived from full genome analysis in Fusaro et al. (2017) indicates some level of genetic reassortment between these lineages in the wider viral population. All viruses detected in Italy after February 2017 were descended from a Poland‐like H5N8 virus dated 16 October 2016 (95% HPD, 7 Sep‐29 Nov). The second wave of the epidemic was comprised of two genetically distinct groups (labelled ‘Italy‐A’ and ‘Italy‐B’ in Figure 1b following previous work (Mulatti et al., 2018)). Black diamonds labelled ‘A’ and ‘B’ in Figure 1b indicate the nodes in the tree, with high levels of posterior support, at which Italy‐A and Italy‐B groups can be confidently defined. Molecular clock dating indicates the time of the node defining Italy‐A as being 8 March 2017 (95% HPD, 26 February‐8 May) and Italy‐B as being 20 May 2017 (95% HPD, 4 April‐2 July). As a result, we can be confident that the ancestors of the Italy‐A and Italy‐B lineages had diverged prior to the first case of the second wave, detected on 19 July, indicating that there were at least two epidemiological origins to the second wave of cases.

### 3.1 Phylogeographic reconstruction of dispersal history in continuous space

To investigate diffusion dynamics during the course of the epidemic, geographical coordinates associated with cases were integrated into phylogenetic analysis using the continuous phylogeography approach described by Lemey et al. (2010). A homogenous Brownian diffusion model of geographical spread was compared with relaxed random walk (RRW) models, which involve branch‐specific rates of virus diffusion drawn from an underlying distribution (Cauchy, gamma or lognormal). Each of the RRW models was strongly favoured over the homogenous Brownian diffusion model (difference in AICM > 100) indicating strong evidence for different rates of virus spread across the phylogeny. The RRW model with branch rates drawn from a Cauchy distribution was consistently favoured over the more flexible RRW models where branch‐specific rates were drawn from either a gamma or lognormal distributions.

**Figure 2:**
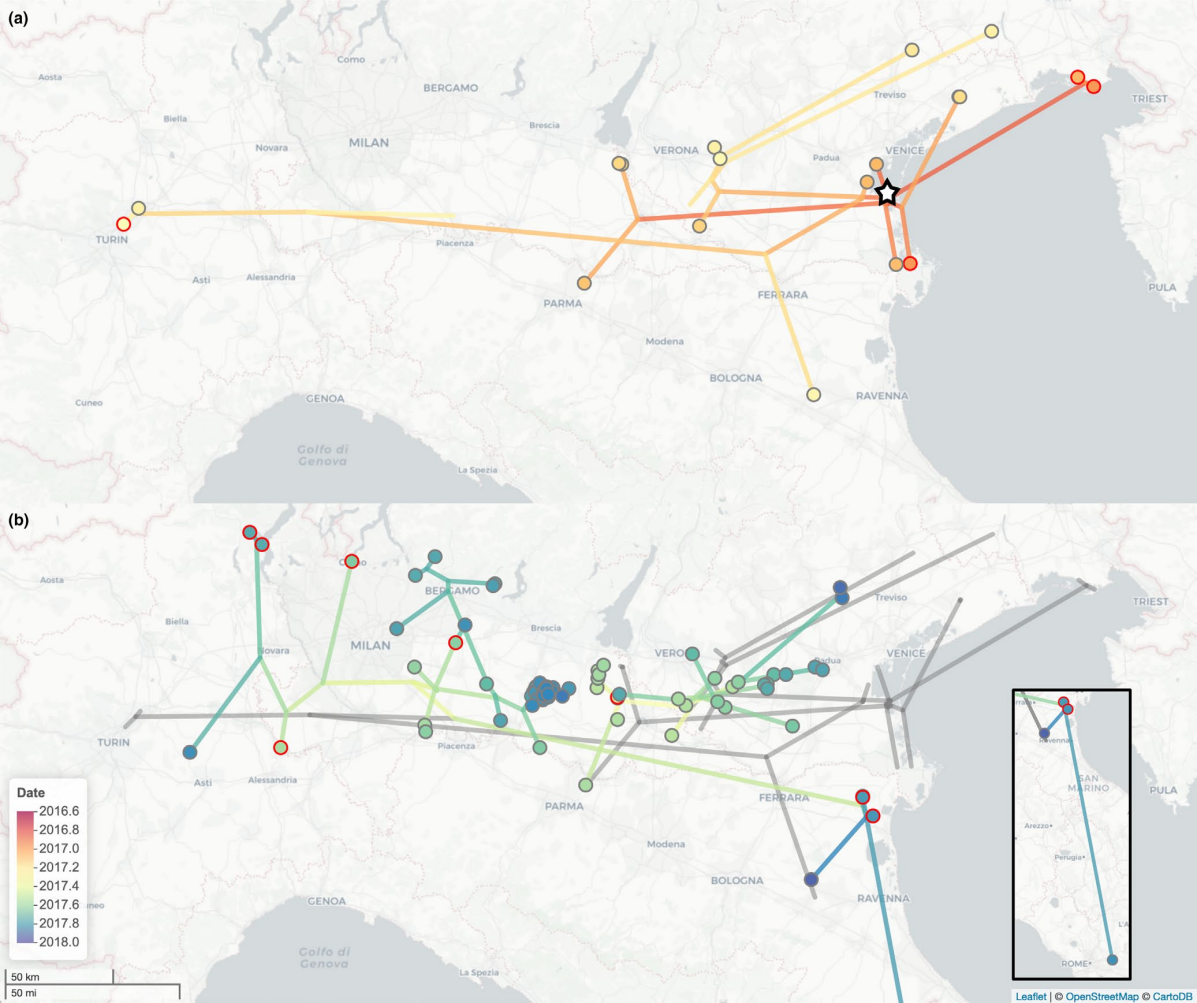
Phylogeographic reconstruction of 2016–17 influenza A(H5N8) epidemic in Italy. Time‐scaled MCC phylogenetic tree shown in geographical space coloured by date. Sampled viruses are indicated as belonging to domestic cases (grey rings) or wild birds (red rings). (a) The phylogeny linking wave 1 cases plotted in colour. A black and white star marks the estimated location of the root. (b) The phylogeny linking wave 2 cases plotted in colour on top of the wave 1 phylogeny in grey. Inset shows the transmission leading to a single case in the Lazio region. Map tiles by Carto, under CC BY 3.0. Data by OpenStreetMap, under ODbL

A spatiotemporal reconstruction of the epidemic is shown in Figure 2, with the MCC tree plotted in geographical space, with separate panels for the first (Figure 2a) and the second epidemic waves (Figure 2b, Italy‐A and Italy‐B genetic groups in Figure 1) to aid visualization. Figure 2a shows the first wave with a likely point of entry and early spread in coastal areas near to the Venetian lagoon, followed by spread generally restricted to eastern areas with one long‐distance westwards movement leading to two cases near Turin (one wild bird and one domestic). In Figure 2b, there is a west–east geographical separation with Italy‐A restricted to the east and Italy‐B tending to remain in the west, though with a long‐distance movement leading to a small cluster of wild bird and domestic cases near the coast to the east of Ferrara. From this cluster, a southwards dispersal leading to a single domestic case in the Lazio region is inferred (shown in inset Figure 2b). Uncertainty in both the tree and reconstructed geographical locations is visualized in Figure S1.

Geographical coordinates and times associated with nodes of the phylogenetic trees in the posterior sample were used to calculate the distance and timing of reconstructed movements. Across a posterior sample of 100 BEAST trees, a highly right‐skewed distribution of distances of reconstructed movements had a mean of 19 km, a median of 4.0 km and a long right tail with a maximum of 405 km. This indicates the epidemic was dominated by local movements of shorter distance and interspersed with rarer long‐distance movements. A histogram showing the distribution of dispersal distances associated with branches of the MCC tree broken down into wave 1, wave 2 Italy‐A and wave 2 Italy‐B is shown in Figure S2. Reconstructed movements for wave 1 were generally of greater distance (mean = 33 km, median = 11 km) than wave 2 (mean = 11 km, median = 2.5 km). Within wave 2, the Italy‐A lineage had a lower mean distance (7.6 km) though similar median distance (2.3 km) compared with Italy‐B (mean = 14 km, median = 2.6 km). This reflects the distribution of movements associated with Italy‐B being particularly right‐skewed with many very short movements associated with local spread and occasional very long‐distance dispersal events.

### 3.2 Quantification of rates of transition between geographical regions

**Figure 3:**
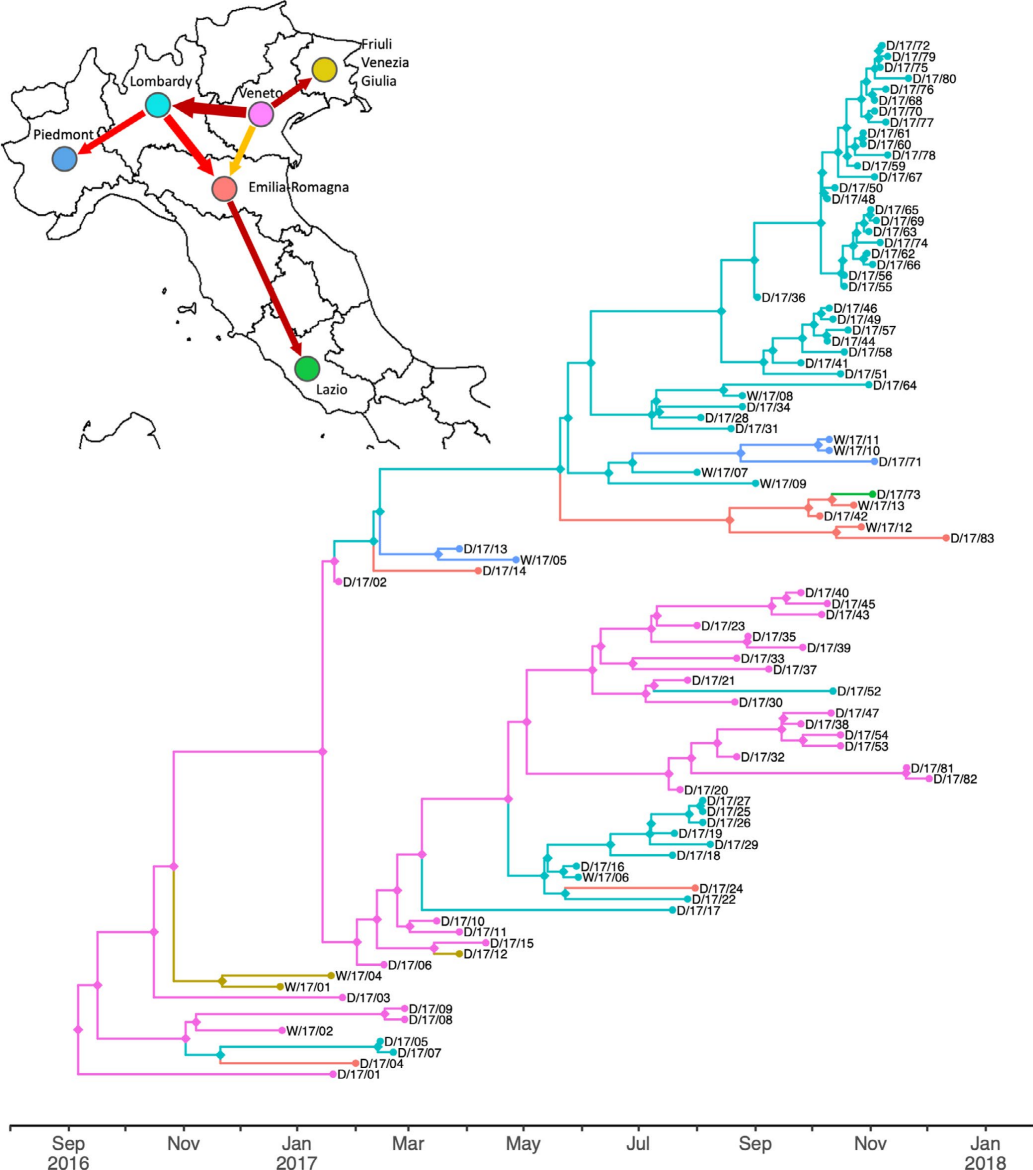
Dynamics of region‐to‐region transmission from discrete trait analysis. Time‐scaled maximum clade credibility HA phylogeny with branches coloured according to the region inferred for the descendant node following the colour scheme of the network map (top‐left) which shows phylogenetically robust region‐to‐region transitions. The width of arrows indicates the rate of transition occurrence (the number of transitions per unit of time), and arrow colour indicates posterior support: dark red indicates inclusion probability 0.9–1.0; red, 0.8–0.9; dark orange, 0.7–0.8; light orange, 0.6–0.7; yellow, 0.5–0.6

Transition rates between the six geographical regions (Veneto *n* = 28, Emilia‐Romagna *n* = 7, Friuli‐Venezia Giulia *n* = 3, Lazio *n* = 1, Lombardy *n* = 51 and Piedmont *n* = 5) where cases were detected during the course of the epidemic were also estimated using a model of discrete trait evolution. Of the 30 possible region‐to‐region transition rates, BSSVS indicated that six were significant using a 0.5 inclusion probability as a cut‐off (Figure 3; inclusion probabilities, Bayes factor support and conditional transition rates for all transitions are shown in Table S3). The greatest rate of region‐to‐region transmission across both waves of the epidemic was identified as being from Veneto to neighbouring Lombardy. Veneto was also identified as the most probable location of the most recent common ancestor of the sampled viruses (posterior probability = 0.99), consistent with the continuous phylogeographic reconstruction.

**Figure 4:**
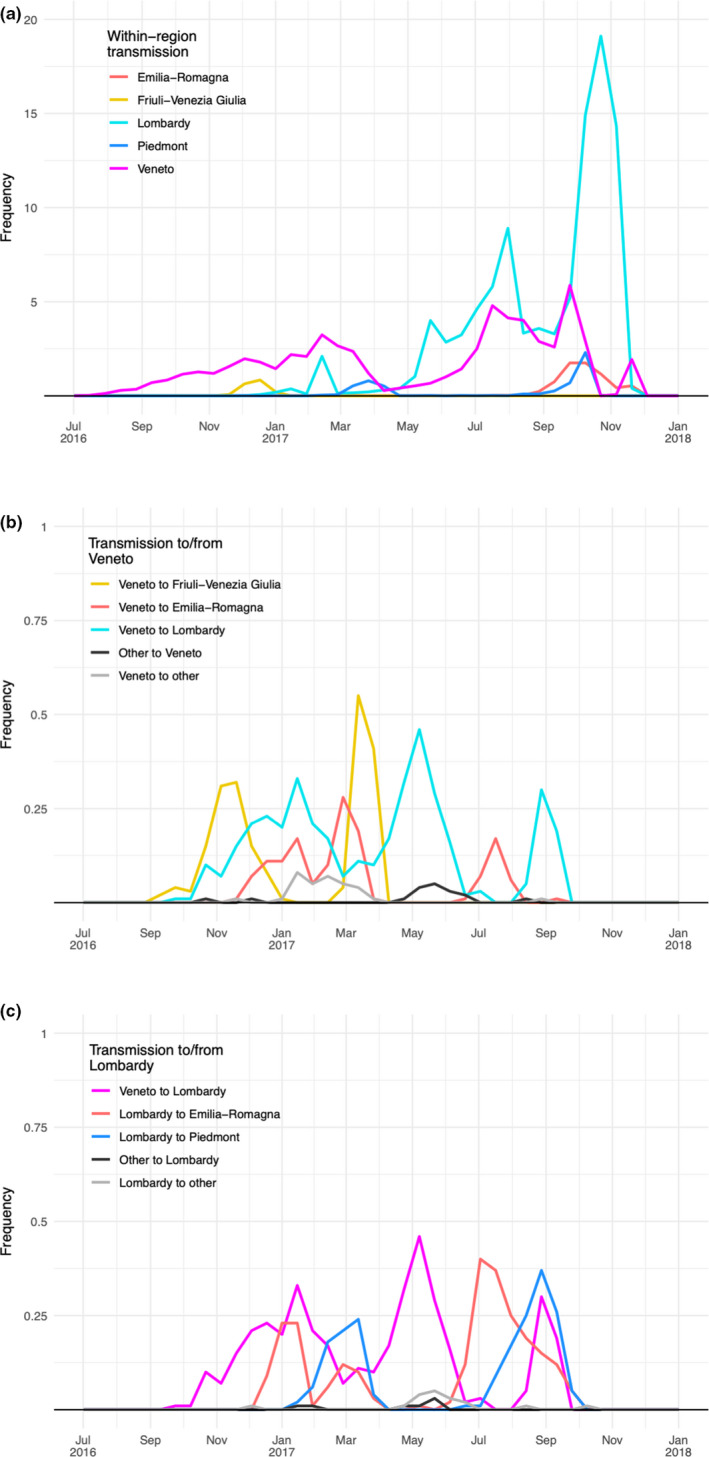
Timing of transmission within and between geographical regions. (a) Rates of within‐region transmission through time. (b) Rates of transition into and out of the Veneto region. (c) Rates of transition into and out of Lombardy though time. All rates are averaged across a posterior sample of 100 trees

To examine the rate of transmission between and within regions through time, dates and inferred ancestral regions were extracted from a sample of 100 trees from the BEAST posterior. Distributions of within‐region transmission are shown in Figure 4a. Distributions of transitions in and out of Veneto and Lombardy, the two most connected regions are shown in Figure 4b,c, respectively. These distributions account for uncertainty in phylogeny, inference of ancestral states and dates associated with internal nodes. Within‐region transmission in 2016 is restricted to Veneto and Friuli‐Venezia Giulia. Transmission within Veneto is inferred to continue at a limited level throughout 2017. Inter‐region transmission events are estimated to have been relatively rare and uncertainty in the timing of such events means that the fortnightly rates of transmission in 4b and 4c are below one. Early transmission from Veneto to Friuli‐Venezia Giulia and Lombardy is estimated as most likely having occurred in late December 2016 or early January 2017. This is followed by likely onwards transmission from Lombardy to Emilia‐Romagna and then to Piedmont. Further transmission from Veneto is detected to Friuli‐Venezia Giulia in March 2017, and then, to Lombardy. Figure 4a shows within‐Lombardy transmission then begins to rise from May 2017 reaching a first peak in July–August (examination of the phylogeny in Figure 3 indicates this is largely Italy‐A) followed by a higher peak between late September and early November (largely Italy‐B). Transmissions from Lombardy to other regions during the second half of 2017 are inferred to have occurred prior to this peak in within‐Lombardy transmission: firstly, to Emilia‐Romagna most probably in July, and secondly, to Piedmont most probably in August (Figure 4c). Additionally, a phylogenetically robust transmission from Emilia‐Romagna to Lazio was detected (Figure 3), and a long‐distance southwards dispersal of roughly 330 km (Figure 2b) estimated to have occurred between 11 October (95% HPD, 23 September‐22 October) and 2 November 2017, the date of sampling for the Lazio case (D/17/73).

### 3.3 Quantification of the role of wild birds in transmission between host types

To explore transmission dynamics at the wildlife–domestic interface and the relative roles of primary incursions into the domestic sector from wild birds and secondary spread between domestic premises, rates of transmission between host types were investigated. Six categories of host type that accounted for differences in species and production type were considered (backyard *n* = 21, broiler *n* = 5, layer *n* = 12, turkey *n* = 39, domestic waterfowl *n* = 6 and wild bird *n* = 12). Due to the nature of current surveillance practices, it is likely that infected wild birds were significantly under‐sampled, relative to domestic birds, during the H5N8 epidemic. Modelling host type as a discrete phylogenetic trait (Lemey et al., 2009) has been shown to produce biased results when populations differ markedly in sampling intensity, underestimating the rate of transmission from the under‐sampled population to other populations and overestimating rates of transmission to the under‐sampled population (Dudas et al., 2018). This bias would potentially overestimate the proportion of domestic hosts at internal nodes of the phylogeny, inflate transmission rates from domestic to wild birds and deflate rates from wild to domestic birds. To account for the under‐sampling of wild birds, we used phylogenetic distances between domestic cases averaged across time‐measured phylogenies to infer the presence of unobserved, cryptic wild bird‐mediated transmission across the phylogeny. This inference was used to adjust the results of the discrete trait analysis such that host types were retained at internal nodes only when distances between domestic cases did not indicate likely transmission in wild birds.

In spite of the potential for bias described above, the most probable host type at the root of the tree was inferred to be wild bird, albeit with considerable uncertainty (posterior probability = 0.63), and rates of wild bird to domestic transmission were estimated to be higher than rates of transmission from domestic to wild birds. Nonetheless, the results of the discrete trait model contradict other sources of information (Mulatti et al., 2018) and resulted in only 21% of internal nodes being estimated to be wild bird (full results of discrete trait analysis shown in Figure S3 and Table S4). To adjust for under‐sampling of the epidemic in wild birds, patristic distances between domestic cases were calculated across the posterior set of time‐scaled HA phylogenies and each domestic case was assigned a putative domestic ancestor that was the phylogenetically closest domestic case recorded at an earlier date. Evolutionary distances between domestic cases and putative domestic ancestors ranged between 0.03 and 0.70. These bounds are interpreted as typifying secondary (domestic to domestic) and primary (wild bird to domestic) transmission, respectively. Higher evolutionary distances between domestic cases were assumed to indicate wild bird‐mediated transmission, even in the absence of wild bird sequences positioned nearby in the tree. Phylogenetic distances within the observed range were used as thresholds to infer host type at each internal node of the phylogeny, with higher threshold values resulting in a lower proportion of internal nodes being inferred to be wild bird.

**Figure 5:**
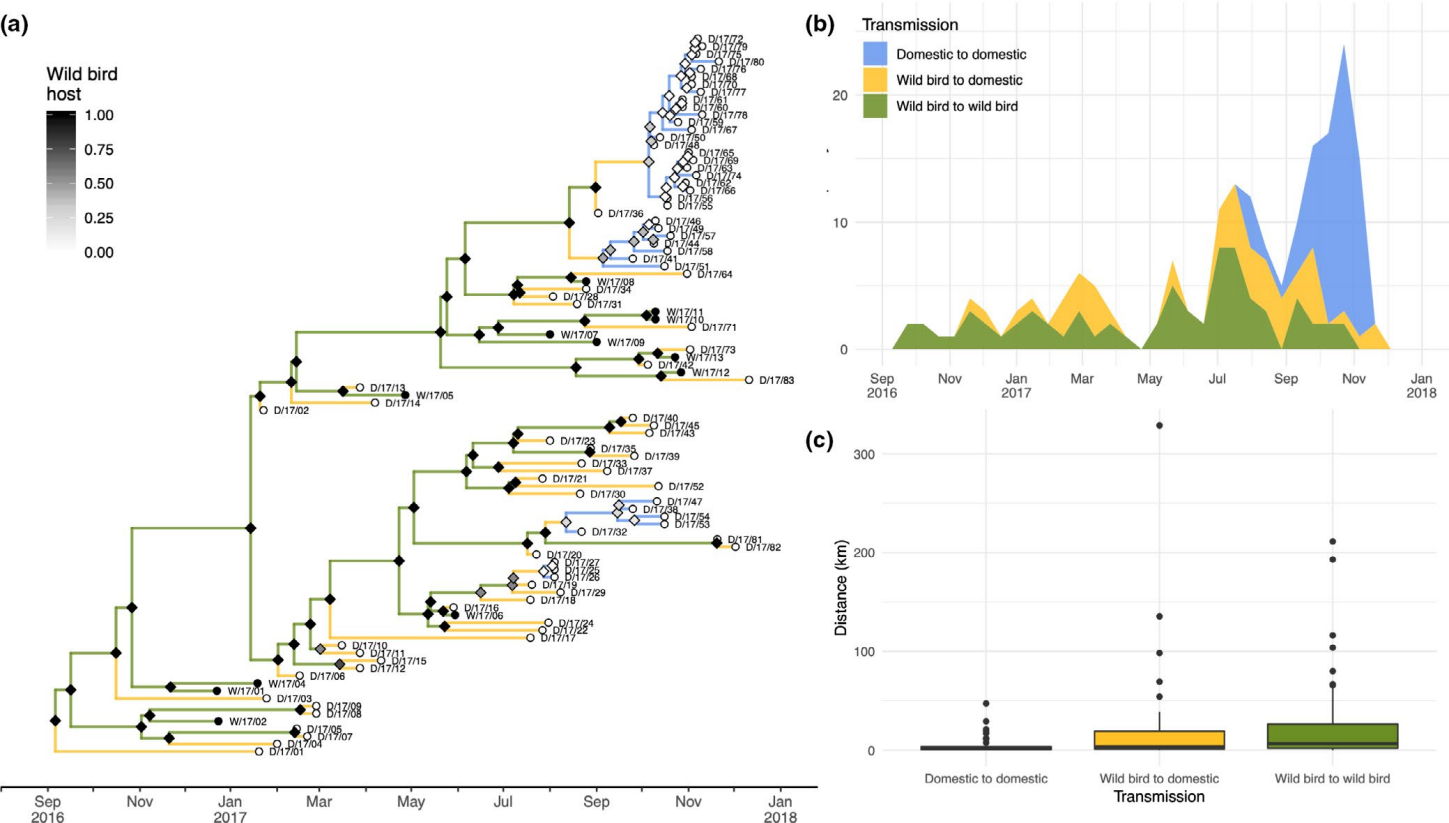
Inference of cryptic wild bird‐mediated transmission. (a) Phylogeny with inferred host type at internal nodes (diamonds) coloured by proportion of reconstructions for which wild bird was inferred across a range of evolutionary distance threshold values, according to greyscale legend. Tip points (circles) are also coloured to indicate whether case was a wild (black) or domestic (white) bird. Branch colour indicates whether associated transmission is inferred to be wild bird to wild bird (green), wild bird to domestic (yellow) or domestic to domestic (blue) on the basis of internal nodes inferred using optimal evolutionary threshold. (b) Stacked distribution of times of transmission within and between wild and domestic birds displayed through time in fortnightly bins. (c) Boxplot showing relationship between per‐branch transmission type and dispersal distance

Domestic cases were classified as resulting from primary or secondary transmission, according to host type (wild or domestic) inferred at internal nodes using a range of threshold phylogenetic distances. The resulting classification was compared with the classification of cases made using established epidemiological methods (Mulatti et al., 2018). A threshold value of 0.19 was found to provide a good level of accordance with existing methods, balancing sensitivity and precision and maximizing an *F*‐score calculated with a beta value of 1.5 (Table S5), and was applied across the phylogeny. Using this threshold, each branch of the phylogeny was classified as representing either transmission within wild birds, from wild birds to domestic, or domestic to domestic. Transmission type is shown mapped to the phylogeny in Figure 5a and through time in Figure 5b. Using a reduced range of threshold values (0.115–0.285), which resulted in a reasonable level of accordance with published estimates, the proportion of threshold values at which internal nodes of the phylogeny ancestral hosts were estimated to be a wild bird was calculated. In Figure 5a, internal nodes of the phylogeny are shaded according to these proportions indicating the degree to which inferred transmission type is robust with regard to threshold value. This shows that at most thresholds considered, secondary transmission between domestic premises was restricted to the second epidemic wave (July to December 2017). Multiple clusters of phylogenetically linked secondary domestic cases were inferred under a range of evolutionary thresholds, though the number of distinct clusters varied according to the choice of threshold.

**Figure 6:**
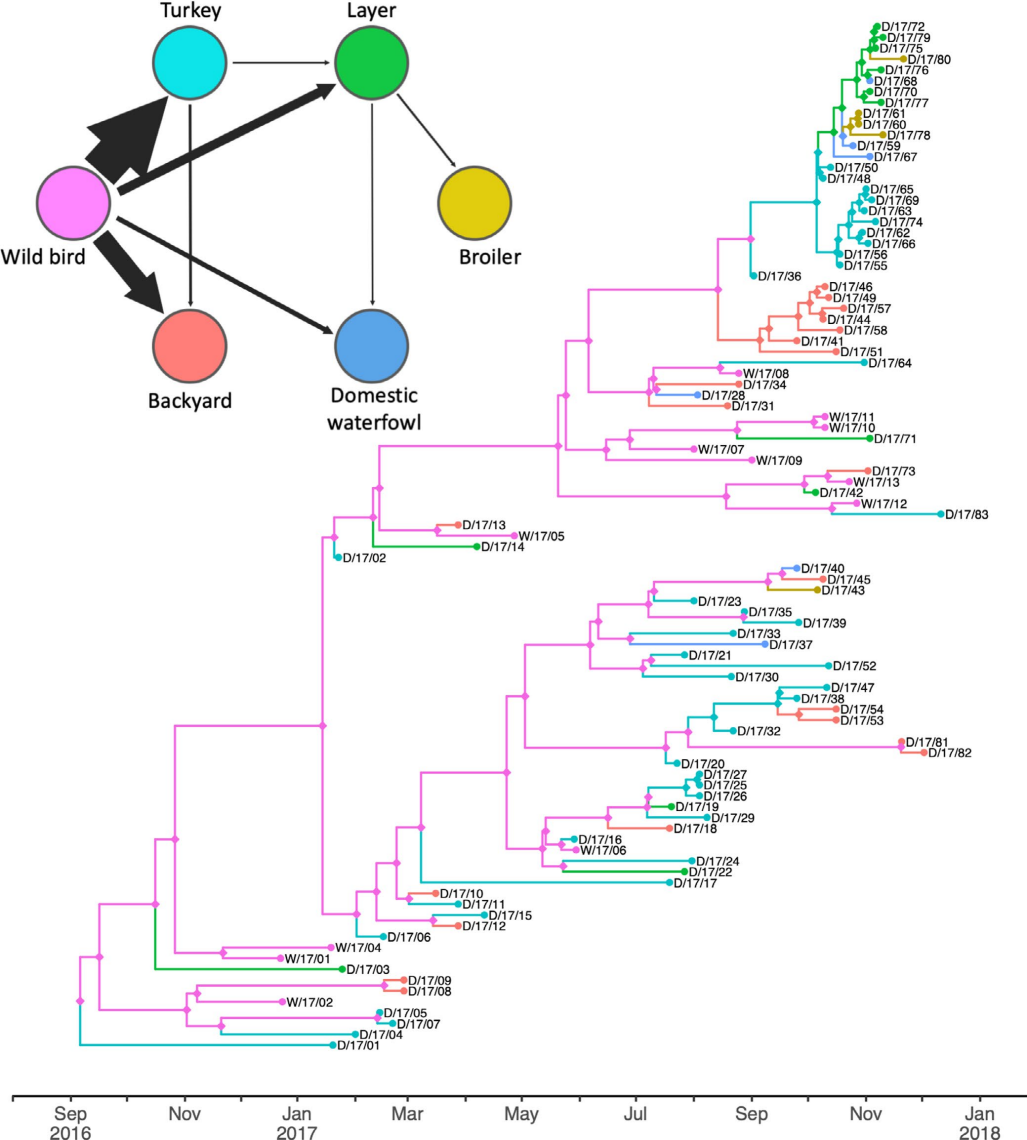
Host‐type transmission dynamics Time‐scaled maximum clade credibility HA phylogeny with branches coloured according to the host type inferred for the descendant node following the colour scheme of the network map (top‐left) which shows phylogenetically robust host‐to‐host transitions. Host type at internal nodes reconstructed using discrete traits model with BSSVS and adjusted to infer the presence of unobserved wild bird hosts using phylogenetic–temporal distances between domestic cases. In the network map, phylogenetically robust transitions are shown with arrow width relative to the rate of transition occurrence (the number of transitions per unit of time)

The phylogenetic tree with host types reconstructed at internal nodes and adjusted for the presence of cryptic wild bird‐meditated transmission is shown in Figure 6, alongside a network map showing significant rates averaged across a posterior sample of trees. The highest rate of transmission between host types was estimated as being from wild birds to domestic turkeys, followed by the rate of wild birds to backyard premises. There is also robust evidence of transitions from wild birds to domestic waterfowl and laying chickens, but not to broiler chickens (though a single wild bird to broiler transition is inferred in the MCC, Figure 6). Of the five domestic host categories, phylogenetically robust onwards transmission to at least one other production type was detected from turkeys and from laying chickens. Across a posterior sample of trees, there was no robust evidence of onwards transmission to other production types from either backyard, domestic waterfowl or broilers. The phylogeny in Figure 6 allows the production types involved in the clusters of domestic to domestic transmission in Figure 5 to be elucidated. The large cluster of secondary cases in the Italy‐B genetic group positioned at the top of the phylogeny included turkeys, layers, domestic waterfowl and broilers, with turkeys being the most likely primary host in this cluster. Secondary transmission is also inferred within a smaller cluster of Italy‐B cases that affected only rural backyard premises with no evidence of transmission to other production types. Within the Italy‐A genetic group, there was support for secondary transmission within a cluster of turkey farms including cases D/17/25, D/17/26 and D/17/27, which were located in western Lombardy. A second cluster of cases inferred to result from transmission within domestic birds included cases in turkey farms and two backyard premises. Examination of epidemiological information associated with these cases indicated a lack of contacts between the affected farms, suggesting they could be linked through a common source of infection, perhaps involving a wild bird population with particularly high levels of contacts to domestic premises.

The spatial pattern of host type‐to‐host type transmission was visualized by projecting the phylogeny in Figure 5a in geographical space (Figure S4). Wild bird driven dispersal can be seen to be responsible for geographical expansion of the epidemic. Wild bird to domestic transmission was particularly prevalent in the eastern portion of the area affected during the epidemic, occurring more frequently during first wave of cases and within the Italy‐A genetic sub‐epidemic during the second wave. The large cluster of domestic to domestic transmission in the Italy‐B genetic group (Figure 5a) is restricted to a small geographical area to the south of Brescia (Figure S4). In contrast, the smaller cluster of domestic to domestic transmission in the Italy‐B genetic group covers a significantly larger area to the east of Milan, extending north towards a number of backyard cases in the vicinity of Bergamo.

To investigate the role of different host transmission types in virus dispersal dynamics, we also examined the relationship between reconstructed host type and virus dispersal distances (Figure 5c). Inferred transmission type explained significant variation in mean dispersal distance (general linear model, *p* < .005) with both intrawild bird (23 km) and wild bird to domestic transmission (23 km) tending to occur across greater distances than domestic to domestic transmission (4 km). As expected, most dispersal events associated with transmission within domestic birds covered relatively short distances (*Q*_3_ = 3.38 km), and however, a number of longer‐distance outliers were identified including three movements inferred to cover distances greater than 20 km with a maximum of 47 km. Closer inspection revealed all three of these longer distances belonged to cluster of domestic to domestic transmission covering an area to the east of Milan connecting backyard cases in the Italy‐B genetic group.

## 4 DISCUSSION

Integrated phylogenetic analyses of genetic, geographical and host data allowed us to reconstruct the spatiotemporal structure and characterize the dispersal processes underlying the 2016–17 HPAI A(H5N8) epidemic in Italy. This analysis structured around a time‐aware phylogenetic reconstruction allowed us to co‐infer dispersal times and distances as well as the host types involved in such movements. Faced with an under‐sampled virus reservoir in wild birds, we have described a phylogeny‐based approach to infer the presence of cryptic wild bird‐mediated transmission. This approach identifies areas of the phylogeny where viruses were not sampled from wild birds but where phylogenetic–temporal distances between domestic cases indicate potential incursions from the wild bird population rather than secondary transmission between domestic premises. This analysis indicated the importance of wild birds, in spite of the relatively low number of observed wild bird cases, in both epidemic persistence and geographical dispersal. In general, transmission within wild birds is found to explain long‐distance virus movements, while clusters of transmission within domestic hosts are associated with shorter distance local movements.

Ancestral traits associated with the root of the phylogeny indicated the most recent common ancestor of the sampled viruses to be associated with wild birds somewhere in the region of the Venetian lagoon in the second half of 2016 (95% HPD, 28 July–4 November). This is consistent with the timing of arrival of migratory birds in north‐eastern Italy (Spagnesi & Serra, 2005), indicating the potential introduction of the A(H5N8) HPAI virus from eastern Europe. Recorded cases fell into two distinct epidemic waves. The Veneto region acted as a point of entry for the epidemic in Italy with observed transmissions to Friuli‐Venezia Giulia and to Lombardy from where further dispersal to Emilia‐Romagna and Piedmont was detected during the first epidemic wave (December 2016 to May 2017). Phylogenetic and temporal distances between domestic cases during the first epidemic wave indicated several primary incursions into domestic production from wild birds with no evidence of secondary transmission between domestic premises. A time‐scaled phylogenetic analysis revealed that the two genetic groups that circulated during the second epidemic wave, Italy‐A and Italy‐B, had likely diverged prior to the start of the second wave. The Italy‐A sub‐epidemic was maintained in Veneto, with sparse spill‐over into both Emilia‐Romagna and eastern Lombardy where some further transmission is observed. Only one wild bird was detected within the Italy‐A group (W/17/06), and however, phylogenetic and temporal distances suggest persistence of this genetic group in wild birds with several primary incursions, and some cases that could have resulted from secondary transmission, mainly in fattening turkeys in eastern Lombardy region (Mantua province), and in turkeys and rural farms in Veneto (Vicenza province). The Italy‐B sub‐epidemic appeared to have originated in southern Lombardy following a westward movement of the virus in wild birds, with the first cases being confirmed in early August 2017 in wild birds, a fattening geese farm and two backyards in western Lombardy. Our analysis based on phylogenetic distances between domestic cases suggests at least two distinct clusters of domestic cases linked by secondary transmission. Relative to the rest of the epidemic, Italy‐B was found to have a higher reconstructed rate of domestic to domestic transmission.

Discrete phylogeography allowed phylogenetically robust region‐to‐region transmission routes to be identified, which was useful given control measures are often implemented at the region level. Rates were monitored through time enabling the evaluation of the effectiveness of introduced control measures. For example, the functional separation of the regions Lombardy, Veneto, Piedmont and Emilia‐Romagna was re‐introduced on 28 July 2017, ten days following the detection of HPAI in Lombardy (DGSAF, 2017). Figure 4 indicates lower levels of region‐to‐region transmission after this date despite within‐region transmission rising in the months after July, thus supporting the effectiveness of the enforced restriction and control measures in containing the inter‐regional spread of AI infection. Simultaneous reconstruction of geographical regions and host type allows for inference of the host types involved in dispersal when evaluating control measures. Secondary transmission between domestic premises within the Lombardy region was particularly frequent during the second epidemic wave, and however, there is no evidence of cases in other regions descended from either of the domestic clusters of cases in the Italy‐B clade associated with the region. Instead, the majority of exports from Lombardy appear to have occurred earlier and in association with wild birds. The results of the host‐to‐host transmission analysis corroborated what has been observed in previous epidemics in Italy (Busani et al., 2009; Mannelli, Ferrè, & Marangon, 2006), with fattening turkeys being at higher risk of virus introduction from wild birds, and acting as source of infection for other production types in the cluster of secondary cases. In contrast, chicken broilers accounted for only a small fraction of domestic cases (five out of 83), and in common with backyards and domestic waterfowl, no significant onwards transmission to other production types was detected.

Estimating rates of transmission between under‐sampled reservoir populations and a well‐sampled target species is a common challenge in infectious disease epidemiology. Discrete trait analysis is recognized as tending to underestimate transmission rates from, and overestimate rates towards, under‐sampled populations (Dudas et al., 2018). We have used an approach based on evolutionary distances between domestic cases to infer the presence of cryptic wild bird‐mediated virus transmission in areas of the phylogeny where viruses were not sampled from wild birds. This approach requires some judgement of a threshold distance below which cases can be judged as likely originating from secondary transmission. Given judgements of this kind are already being made by epidemiologists given available spatiotemporal data and knowledge of potential epidemiological links (Mulatti et al., 2018), we see this analysis as providing additional information from genetic data with which the processes driving epidemics can be inferred. This approach also relies on an assumption that the level of domestic to wild bird transmission is low, though this assumption is supported by the discrete trait analysis performed and existing knowledge about the epidemic (Mulatti et al., 2018).

In contrast to the 2016–17 HPAI A(H5N8) epidemic elsewhere in Europe that was generally identified primarily in the wild bird population (Brown, Kuiken, et al., 2017; Brown, Mulatti, et al., 2017), wild bird cases during the epidemic in Italy were relatively rare in comparison with domestic cases (Fusaro et al., 2017; Mulatti et al., 2018). Our analyses indicate the role of wild birds during the Italian epidemic was significantly greater than apparent when considering either the number of wild bird cases or the position of wild bird cases in the phylogeny alone, with wild bird hosts reconstructed at deep nodes associated with long‐distance dispersal events using a standard discrete trait approach. The extent of transmission within wild birds was inferred to be quite stable over the period of the epidemic, which extended beyond the nesting (July/August) and wintering migratory periods (January/February), to periods when migratory movements were limited (Spina & Volponi, 2008). Both the time period spanned and geographical extent of the epidemic, extending far from the large wetlands in proximity to coastal areas, in which domestic incursions associated with migratory birds were initially located (Fusaro et al., 2017; Mulatti et al., 2018), supporting spill‐over from migratory to residential wild birds. Several primary incursions were detected in each of the recognized epidemic waves, whereas secondary spread within the domestic sector was probably restricted to the second wave and peaked in October and November 2017.

Co‐inference of host type and phylogeography enabled the relationship between virus dispersal and host to be investigated. Long‐distance dispersal was far more likely to be associated with branches of the tree inferred to correspond to virus dispersal either within wild birds or transmission from wild birds to domestic, and these movements were responsible for all geographical expansion of the area affected during the epidemic. However, some longer dispersal events were inferred to occur in areas of the phylogeny inferred to representing clusters of secondary spread. These instances represent genetically similar viruses detected on nearby dates that are separated by large geographical distances inconsistent with airborne transmission between domestic premises (Jonges et al., 2015; Scoizec et al., 2018; Torremorell et al., 2016). Therefore, such instances highlight opportunities for further investigation, as they may indicate human‐mediated dispersal, missing domestic cases if the timescale allows, or perhaps undetected domestic–wild bird–domestic transmission. For example, long‐distance virus movements were detected within a cluster of phylogenetically linked rural poultry outbreaks detected from the end of September to the end of October in several provinces of Lombardy region to the east of Milan were identified. In this case, there is knowledge that these geographically diffuse cases seem to be associated with the human‐mediated movement of birds associated with a live bird market. This approach therefore has the potential to detect other instances of human‐mediated virus dispersal. The methods used could be applied in the course of an epidemic, in an almost real‐time fashion, by updating the list of viral sequences to be included in the analyses and the ancillary epidemiological data. This could guide the direction of further investigations in infected farms identified as possible secondary cases, or where genomic distances, and spatiotemporal information could suggest the presence of a common source of infection.

In this paper, we describe the geographical movements and host types underlying the persistence and geographical dispersal of the 2016–17 A(H5N8) HPAI epidemic in Italy. We detect significant heterogeneity in the distances covered and tempo of virus dispersal across the epidemic associated with the host types involved. In addition to reconstructing the pattern of movement across the area affected by the epidemic, we co‐estimate the timing and host types producing inferences that can be particularly useful for evaluating the effectiveness of control measures introduced or to inform the design of future measures. Using a phylogeny‐based approach, we identify an important role of wild birds in the persistence and geographical expansion of the avian influenza epidemic. The role of wild birds is inferred to be much greater relative to the rate at which they were sampled, indicating that increased surveillance in wild birds could enable transmission to be understood in greater detail. To follow this work and further explain epidemic spread, correlations between variation in reconstructed dispersal distances and velocities and environmental variables including elevation, distance to fresh water, land coverage and poultry density are currently being investigated using recently developed methods (Dellicour et al., 2016; Jacquot et al., 2017).

## ETHICAL APPROVAL

The study did not include any experimentation on animals or humans. Samples were taken from dead birds on infected farms, as part of measures provided by the European Council Directive on Avian Influenza (Council Directive 2005/94/EC).

## CONFLICT OF INTEREST

The authors declare no competing interests.
