# Supplementary figures and images for: Spatiotemporal reconstruction and transmission dynamics during the 2016–17 H5N8 highly pathogenic avian influenza epidemic in Italy
Source: Transbound Emerg Dis. 2019 Dec 1;68(1):37–50. doi: 10.1111/tbed.13420 (PMC8048528; doi:10.1111/tbed.13420)

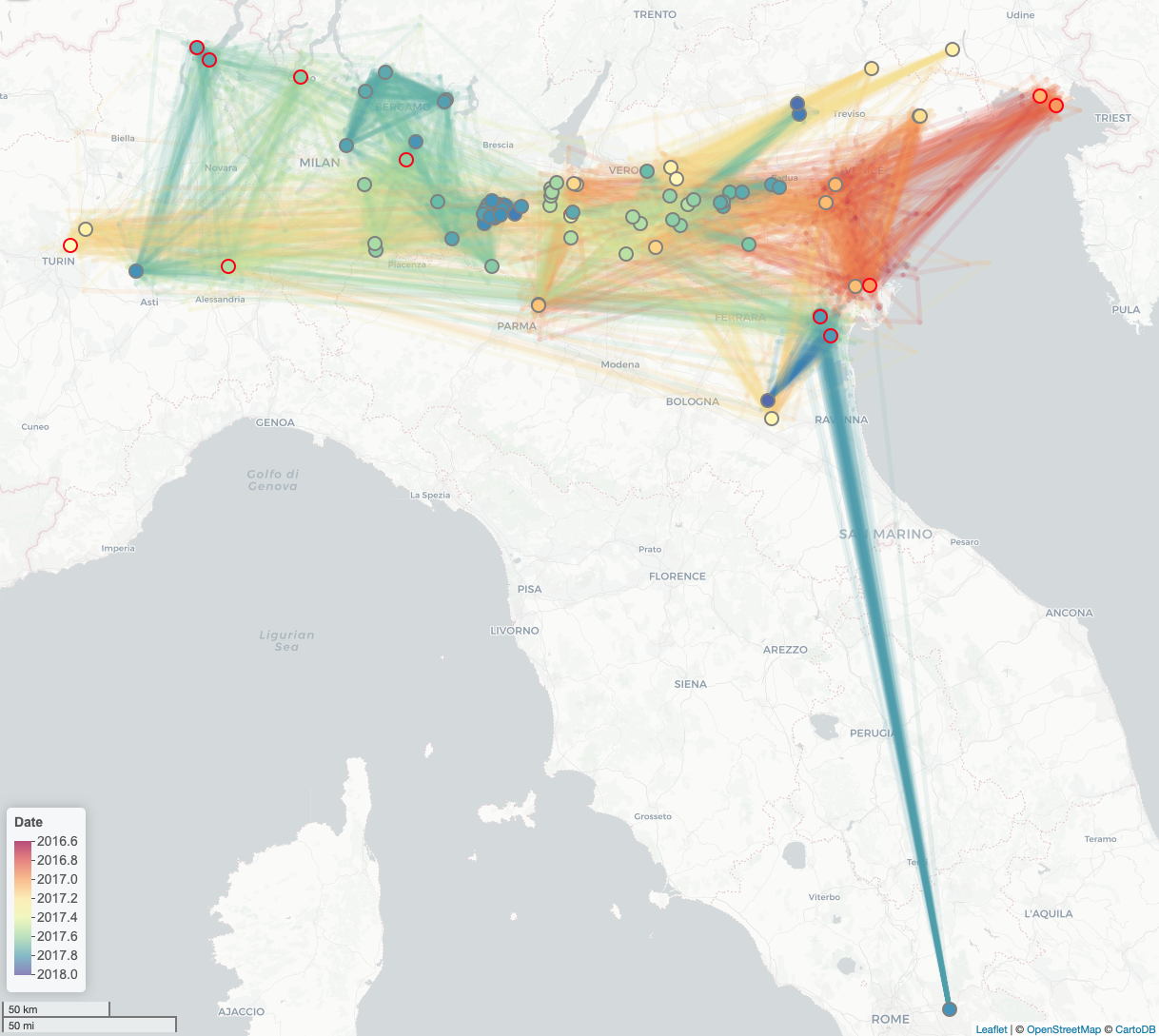

Supplement: Supplementary file 1 [file TBED-68-37-s002.png]

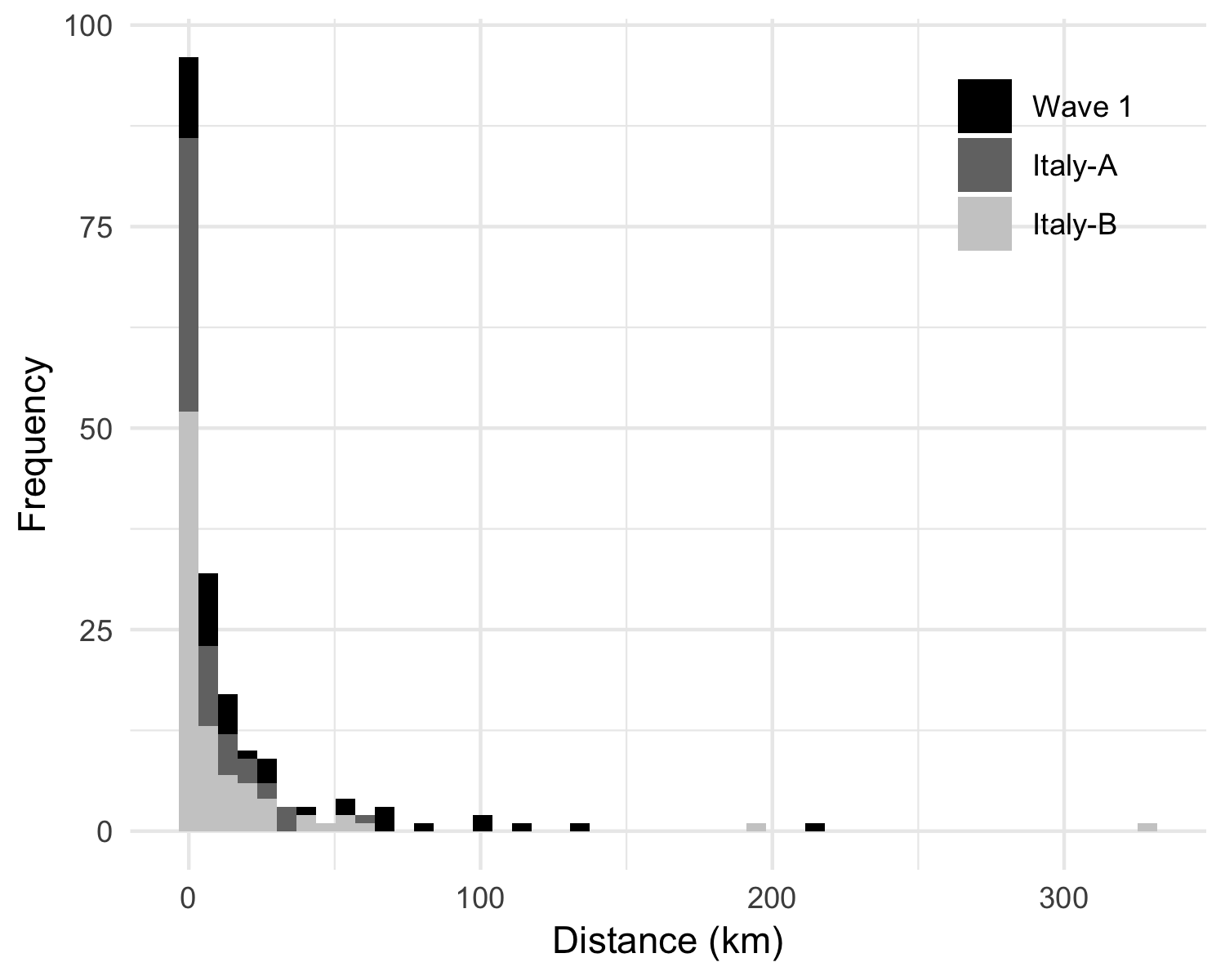

Supplement: Supplementary file 2 [file TBED-68-37-s006.png]

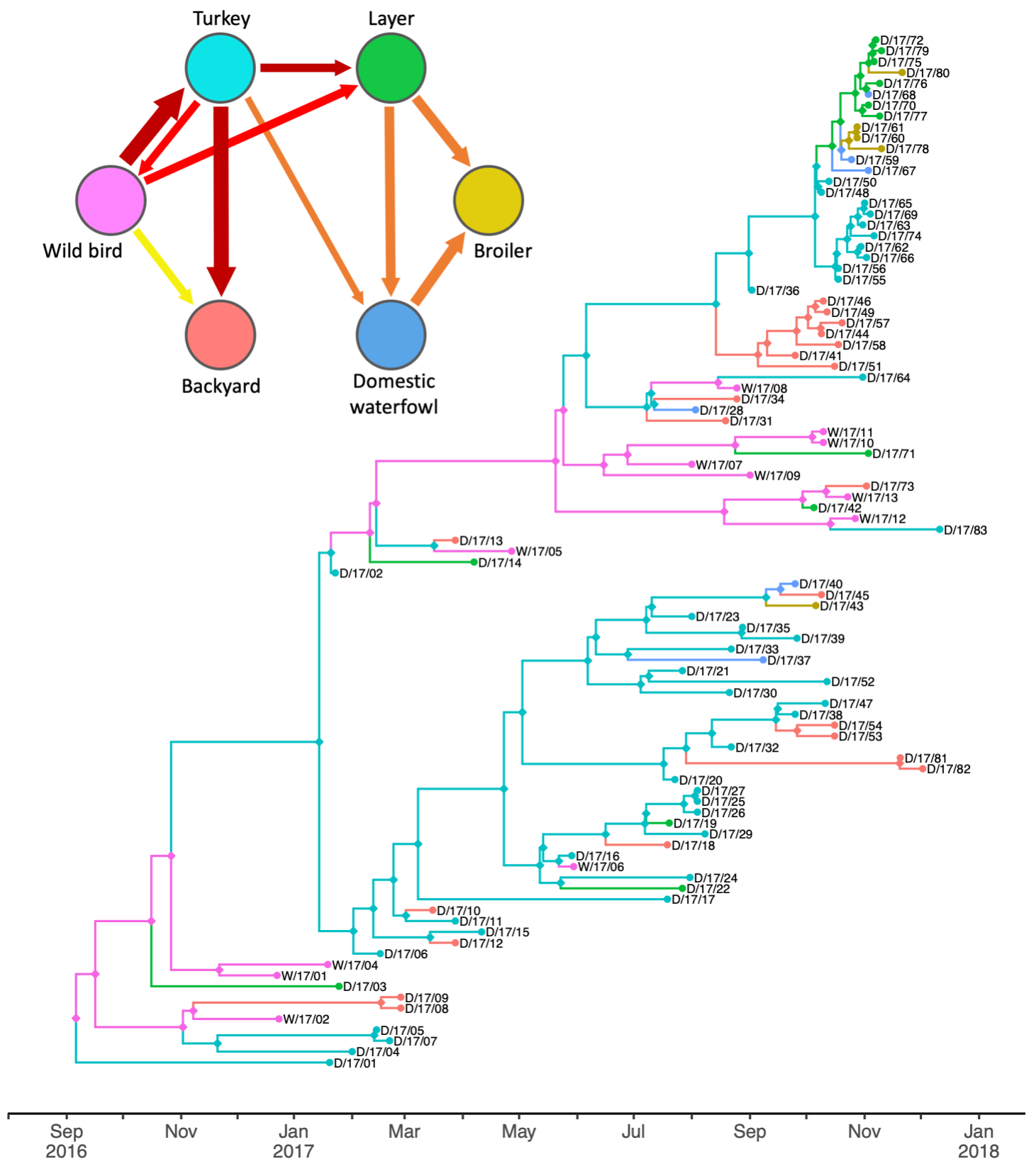

Supplement: Supplementary file 3 [file TBED-68-37-s010.png]

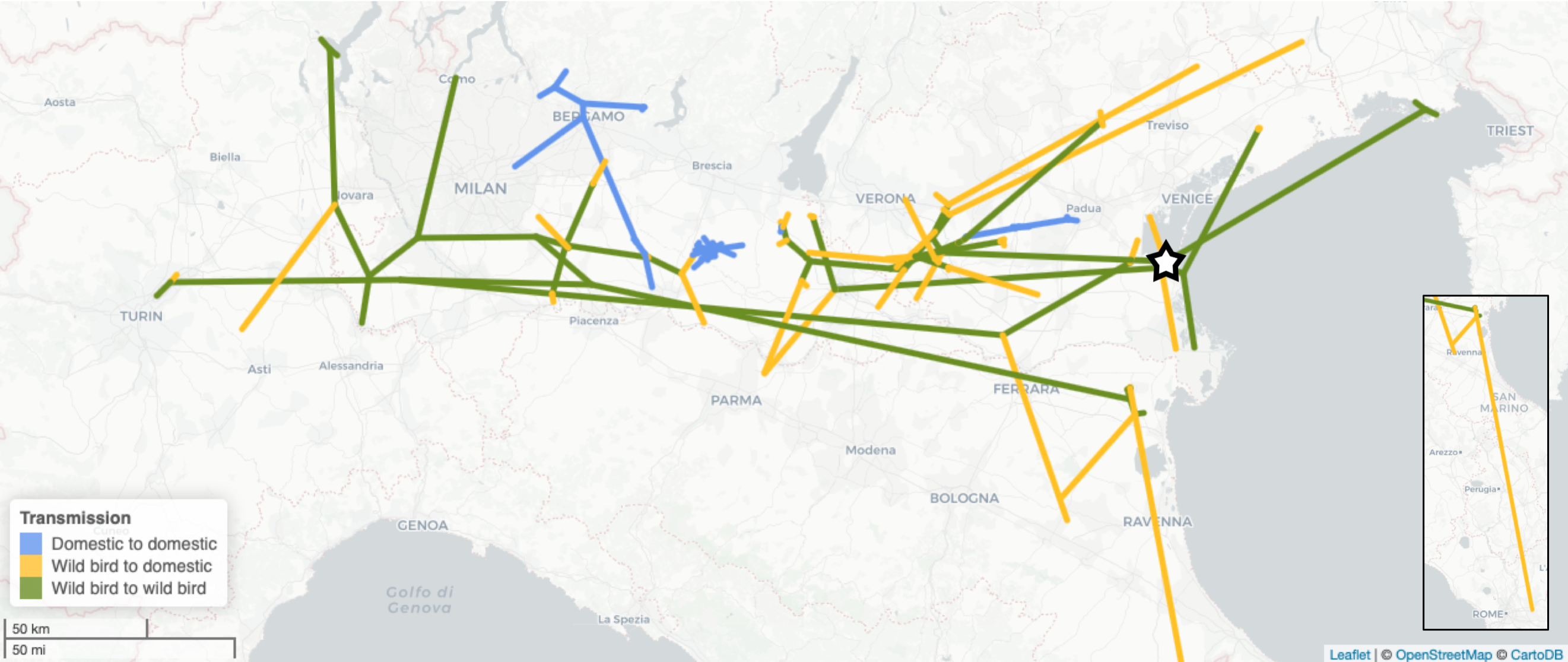

Supplement: Supplementary file 4 [file TBED-68-37-s004.png]
