# Supplementary material for: Spatiotemporal reconstruction and transmission dynamics during the 2016–17 H5N8 highly pathogenic avian influenza epidemic in Italy
Source: Transbound Emerg Dis. 2019 Dec 1;68(1):37–50. doi: 10.1111/tbed.13420 (PMC8048528; doi:10.1111/tbed.13420)
Supplement: Supplementary file 10 [file TBED-68-37-s007.docx]

**Supporting information**

**Figure S1. Posterior uncertainty in phylogeographic reconstruction of 2016-17 influenza A(H5N8) epidemic in Italy.** Superimposition of 100 time-scaled phylogenetic trees sampled from posterior set of trees with times and locations at internal nodes estimated using BEAST. Trees are shown in geographic space coloured by date, according to the legend. Sampled viruses are indicated as belonging to domestic cases (grey rings) or wild birds (red rings). Map tiles by Carto, under CC BY 3.0. Data by OpenStreetMap, under ODbL.

**Figure S2. Histogram showing distribution of great circle distances of reconstructed movements for the maximum clade credibility tree**. Great circle distances calculated using geographic coordinates estimated for internal nodes of the phylogeny using continuous phylogeography in BEAST. Wave 1, wave 2 Italy-A and wave 2 Italy-B are defined following the annotations in Figure 1.

**Figure S3. Ancestral host-types reconstructed using a discrete-traits model**. Time-scaled maximum clade credibility HA phylogeny coloured according to the host type inferred for the descendent node following the colour scheme of the network map (top-left) which shows significant host type-host type transitions inferred using BSSVS. In the network map, arrow width is relative to the rate of transition occurrence (the number of transitions per unit of time) and arrow colour indicates posterior support: dark red indicates inclusion probability 0.9-1.0; red, 0.8-0.9; dark orange, 0.7-0.8; light orange, 0.6-0.7; yellow, 0.5-0.6.

**Figure S4. Phylogeographic reconstruction of 2016-17 influenza A(H5N8) epidemic in Italy showing inferred transmission types.** Time-scaled MCC phylogenetic tree shown in geographic space with branches coloured by inferred transmission type: Domestic to domestic in blue, wild bird to domestic in yellow, and wild bird to wild bird in green. A black and white star marks the estimated location of the root. Inset shows the transmission leading to a single case in the Lazio region. Map tiles by Carto, under CC BY 3.0. Data by OpenStreetMap, under ODbL.

**Table S1. Summary of cases included in study**

**Table S2. Counts of sequences in each discrete state**

**Table S3. Region-to-region transition rates inferred using discrete trait model**

**Table S4. Host type-to-host type transition rates inferred using discrete trait model**

**Table S5. Proportion of internal nodes inferred to be wild bird and identification of secondary cases using a phylogenetic distance-based method with a range of thresholds and using a discrete trait model**
